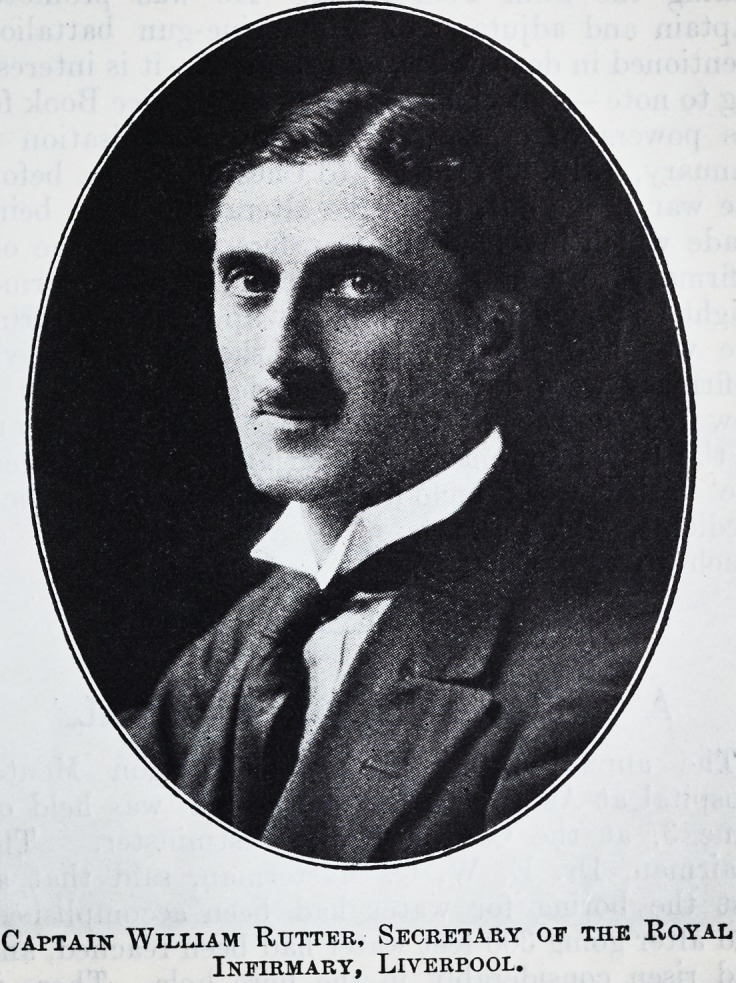# Hospital Men of Mark: Mr. Wade Deacon, C.B.E., and Captain William Rutter

**Published:** 1924-07

**Authors:** 


### HOSPITAL MEN OF MARK.

### MR. WADE DEACON, C.B.E., AND CAPTAIN WILLIAM RUTTER.

The Liverpool Royal Infirmary is fortunate in possessing at the head of its affairs two such experienced and versatile men as Mr. Henry Wade Deacon, C.B.E., the Chairman, and Captain William Rutter, the Secretary. Mr. Deacon is the son of the late Henry Deacon, a partner in the firm of Gaskell, Deacon & Co., formerly one of the great chemical manufacturers, and was educated at King's College, London. He subsequently joined his father in business, but retired in the year 1890, when many of the chemical manufacturers, including Gaskell,

[Elliott & Fry.

MR. H. WADE DEACON, C.B.E., CHAIRMAN OF THE LIVERPOOL ROYAL INFIRMARY.

Deacon & Co., amalgamated and formed the United Alkali Company. Mr. Wade Deacon has always been keenly interested in hospitals and hospital policy. He was chairman of the David Lewis Northern Hospital, Liverpool, from 1902 to 1912, and since then has been chairman of the Liverpool Royal Infirmary. He has therefore had very great experience in hospital management, and is undoubtedly one of the most distinguished hospital experts of the day. As such he has for several years been chairman of the British Hospitals Association. There are, indeed, very few men who make any approach to him in length of experience, or in knowledge of the details of hospital management.

Since Mr. Wade Deacon became associated with the Royal Infirmary he has been successful in maintaining and furthering the reputation of that eminent and old-established institution. Under his chairmanship a ward exclusively for the treatment of tropical diseases has been built and equipped, and an important orthopædic department and an electro-radiograph installation have been added to the Infirmary. The X-ray department has been greatly extended and improved, new gynæcological and obstetrical operating theatres—probably the finest theatres of the day—have been built, owing to the generosity of Mr. Domingo de Larrinaga, and in addition to all this a splendid up-to-date nurses' home, which is most urgently needed, is now in course of erection. Moreover, it is mainly owing to Mr. Wade Deacon's enthusiastic activities that a sum of money sufficient to form the nucleus of the amount required for its construction and equipment

CAPTAIN WILLIAM RUTTER, SECRETARY OF THE ROYAL INFIRMARY, LIVERPOOL.

has been raised. In addition to Mr. Deacon's knowledge of hospital work, he is greatly interested in education and public welfare generally, being Vice-chairman of the Lancashire County Council. He has also for many years been Chairman of the Education Committee of the Widnes Corporation, besides being a county magistrate and chairman of Quarter Sessions, and Chairman of the Liverpool Gas Company for the last twenty years.

When Captain William Rutter succeeded Mr. F. H. Moore as Secretary and General Superintendent, he had gained his hospital experience in the Midlands and in Wales. Born in 1888, Captain Rutter, who, by the way, retired from the army with the permanent rank of captain, joined the staff of the North Staffordshire Infirmary in 1905, and remained there till the spring of 1913, when he became assistant secretary at King Edward VII.'s Hospital, Cardiff. At the former hospital for nine months the assistant secretary and himself were responsible, in the temporary absence of any house governor, for the administration, and during his time there, when Dr. Basil

Rhodes was secretary and house governor from 1910, a sum of £35,000 was raised for structural alterations, which were completed during his tenure of office. The hospital rules were remodelled, and his immediate task was to reorganise the system of book-keeping for drugs and stores. When he went to Cardiff his duties were to keep the financial books and to prepare the balance-sheet.

At the end of a year Captain Rutter left Cardiff to take his next secretarial post at the Chester Royal Infirmary, where, as its first resident secretary, he remained from April, 1914, to February, 1916, when he joined the army. He served for two years continuously in France, to which he went with a commission after a year's service in England, and Ireland during the Sinn Fein rising. He was promoted captain and adjutant of a machine-gun battalion, mentioned in despatches, and received—it is interesting to note—a special mention in his Service Book for his powers of organisation. On demobilisation in January, 1919, he returned to Chester, where, before the war broke out, extensive alterations were being made which involved the transference from the old infirmary to a new wing, in order that the former might be remodelled and brought up to date. During the war half the 240 beds at the Chester Royal Infirmary were devoted to wounded soldiers. It is now five years since Captain Rutter became Secretary of the Royal Infirmary at Liverpool, a centre where the varied work includes the School of Tropical Medicine, which the late Sir Alfred Jones did so much to establish.

## Figures and Tables

**Figure f1:**
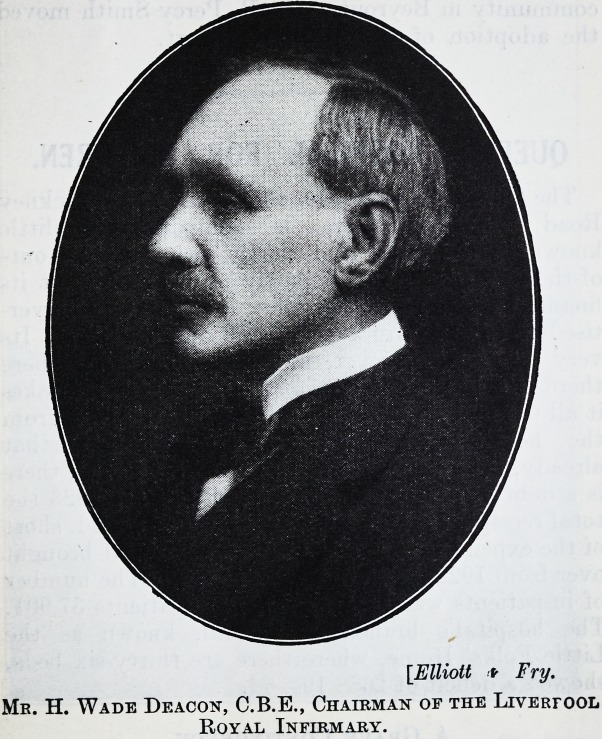


**Figure f2:**